# Increasing but levelling out risk of revision due to infection after total hip arthroplasty: a study on 108,854 primary THAs in the Norwegian Arthroplasty Register from 2005 to 2019

**DOI:** 10.1080/17453674.2020.1851533

**Published:** 2020-11-24

**Authors:** Håvard Dale, Pål Høvding, Sindre M Tveit, Julie B Graff, Olav Lutro, Johannes C Schrama, Tina S Wik, Inge Skråmm, Marianne Westberg, Anne Marie Fenstad, Geir Hallan, Lars B Engesaeter, Ove Furnes

**Affiliations:** a The Norwegian Arthroplasty Register, Department of Orthopaedic Surgery, Haukeland University Hospital, Bergen;; b Department of Clinical Medicine, University of Bergen, Bergen;; c Department of Medicine, Stavanger University Hospital, Stavanger;; d Department of Orthopaedic Surgery, St Olav University Hospital, Trondheim;; e Department of Orthopaedic Surgery, Akershus University Hospital, Lørenskog;; f Division of Orthopaedic Surgery, Oslo University Hospital, Oslo, Norway

## Abstract

Background and purpose — Focus on prevention, surveillance, and treatment of infection after total hip arthroplasty (THA) in the last decade has resulted in new knowledge and guidelines. Previous publications have suggested an increased incidence of surgical revisions due to infection after THA. We assessed whether there have been changes in the risk of revision due to deep infection after primary THAs reported to the Norwegian Arthroplasty Register (NAR) over the period 2005–2019.

Patients and methods — Primary THAs reported to the NAR from January 1, 2005 to December 31, 2019 were included. Adjusted Cox regression analyses with the first revision due to deep infection after primary THA were performed. We investigated changes in the risk of revision as a function of time of primary THA. Time was stratified into 5-year periods. We studied the whole population of THAs, and the subgroups: all-cemented, all-uncemented, reverse hybrid (cemented cup), and hybrid THAs (cemented stem). In addition, we investigated factors that were associated with the risk of revision, and changes in the time span from primary THA to revision.

Results — Of the 108,854 primary THAs that met the inclusion criteria, 1,365 (1.3%) were revised due to deep infection. The risk of revision due to infection, at any time after primary surgery, increased through the period studied. Compared with THAs implanted in 2005–2009, the relative risk of revision due to infection was 1.4 (95% CI 1.2–1.7) for 2010–2014, and 1.6 (1.1–1.9) for 2015–2019. We found an increased risk for all types of implant fixation. Compared to 2005–2009, for all THAs, the risk of revision due to infection 0–30 days postoperatively was 2.2 (1.8–2.8) for 2010–2014 and 2.3 (1.8–2.9) for 2015–2019, 31–90 days postoperatively 1.0 (0.7–1.6) for 2010–2014 and 1.6 (1.0–2.5) for 2015–2019, and finally 91 days–1 year postoperatively 1.1 (0.7–1.8) for 2010–2014 and 1.6 (1.0–2.6) for 2015–2019. From 1 to 5 years postoperatively, the risk of revision due to infection was similar to 2005–2009 for both the subsequent time periods

Interpretation — The risk of revision due to deep infection after THA increased throughout the period 2005–2019, but appears to have levelled out after 2010. The increase was mainly due to an increased risk of early revisions, and may partly have been caused by a change of practice rather than a change in the incidence of infection.

“Postoperative infection is the saddest of all complications…” John Charnley postulated in 1982 (Waugh and Charnley 1990). Despite advances in knowledge and awareness of prophylactic perioperative routines, there are indications that the incidence of infections after total hip arthroplasty (THA) is still increasing (Dale et al. 2012, Parvizi et al. 2013, Lenguerrand et al. 2017, Parvizi et al. 2017, Brochin et al. 2018, Kurtz et al. 2018). To disclose changes in the risk of infection we need a large number of primary THAs, registered in a uniform manner. The Norwegian Arthroplasty Register (NAR) found an increasing risk of deep infection after primary THA during the years 1987–2007. Over 10 years ago, Kurtz et al. (2007) projected a substantial demand for revisions due to infection in the coming decades. We have now assessed changes in the risk of surgical revision due to deep infection for THAs reported to the NAR during the years 2005 to 2019, as a follow-up of our previous study (Dale et al. 2009). In addition, we investigated factors that could be associated with revision, and the time span between primary and revision surgery.

## Patients and methods

Since its inception in 1987, the NAR has registered detailed data on primary THAs and THA revisions. The data gathered includes information on patient characteristics, indication for THA, and surgery-related factors such as approach, type of implant, method of fixation, and duration of surgery. The unique identification number of each inhabitant of Norway is used to link the primary THA to any subsequent revision (Havelin et al. 2000). The data is validated, with 97% completeness of reporting of primary THAs, 93% reporting of revisions, 100% coverage of Norwegian hospitals, and 100% reporting of deaths (Furnes et al. 2019).

Revision due to infection of the implant is defined as the removal or exchange of the whole or parts of the prosthesis, with deep infection reported as the reason for surgery. Isolated soft tissue debridement without the exchange of implant parts was not reported to the register until 2011, and was therefore not included. The surgeon completes the register form immediately after surgery, and the indication for the revision, infection or other, is based on perioperative assessment and evaluation. The diagnosis is not to be corrected in the NAR according to findings in peroperative bacterial samples. Due to this lack of validation, rate of revision due to infection will be only an approximation of the rate of true periprosthetic joint infection (PJI), as defined by Parvizi et al. (2018).

The period of inclusion and observation in this study was January 1, 2005 to December 31, 2019. In this period, the NAR contained data on 116,779 primary THAs. 7,925 (7%) THAs were excluded due to missing information on covariates. 108,854 primary THAs had complete information and were eligible for analyses.

All THAs were followed until their first revision due to infection or revision for other causes after the primary operation, death, or emigration, or until December 31, 2019. Thus, follow-up was 0–15 years. 3 time periods according to the year of primary THA were compared: 2005–2009, 2010–2014, and 2015–2019, with sub-analyses for the different THA fixation methods (all-cemented, all-uncemented, reverse hybrid [cemented cup], and hybrid [cemented stem]).

### Statistics

Survival analyses were performed with Cox regression models, with year of primary THA as the main risk factor and date of revision due to deep infection as the endpoint. Revision hazard rate ratios (HRR) for the 2 later time periods relative to the 1st time period were calculated and presented as an expression of relative risk, with 95% confidence intervals (CI). We adjusted for the following: age (< 45, 45–54, 55–64, 65–74, 75–84, ≥ 85 years), sex, ASA class, indication for the primary THA (osteoarthritis, inflammatory disease, acute hip fracture, complications after hip fracture, complications after childhood hip disease, avascular necrosis of the femoral head, other), surgical approach (anterior, anterolateral, lateral, posterolateral), duration of surgery (< 70, 70–99, 100–129, ≥ 130 minutes), and fixation (cemented, uncemented, reverse hybrid, or hybrid). Revisions due to infection in the case of monobloc THAs were not recorded if no implant parts were exchanged. We therefore adjusted for modularity of the prosthesis in the Cox analyses. In addition, we performed analyses where monobloc THAs (n = 3,936) were excluded. Monobloc implants were predominantly used early in the study period. More than 99.8% of the THA patients received perioperative antibiotic prophylaxis systemically, and for all cemented components antibiotic-loaded bone cement was used.

We used Cox regression analyses, with time period as the stratification factor, to construct cumulative revision curves (1 minus cumulative survival) at mean values of the covariates, and to assess 5-year revision percentages. Analyses with follow-up restricted to 0–5 years for each period were performed, to assess for the effect of differences in follow-up. In addition, analyses were performed without THAs with metal-on-metal articulations (n = 300), since metal debris reactions may mimic infection. Further, we performed sub-analyses on THAs due to osteoarthritis only (n = 83,770), as a more homogenous subgroup. We also performed separate Cox analyses on revision due to aseptic loosening as endpoint for all THAs, to be able to compare these with our findings of revision due to infection.

Revision HRR due to infection as a function of year of the primary THA was studied, to give a graphical display of the relationship based on a generalized additive model for survival data (Hastie and Tibshirani 1990). These curves are presented with 95% CIs.

HRRs were calculated for the different potential risk factors for the whole 15-year period adjusted for year of primary THA, to adjust for time-dependent confounding.

The analyses were performed in accordance with the guidelines for statistical analyses of arthroplasty register data (Ranstam et al. 2011a and b). The proportional hazard assumptions of the Cox survival analyses were not completely fulfilled between the 3 time periods when tested by smoothed Schoenfeld residuals (see Figure 3). We therefore assessed the risk of revision due to infection 0–30 days, 31–90 days, 91 days–1 year, and 1–5 years postoperatively.

A study from the Swedish Knee Arthroplasty Register has found that potential overestimation of incidence of revision through the effect of competing risks (death and revision) is negligible, and that Cox analyses are better than competing risk analyses in estimating revision risks in arthroplasty register data (Ranstam and Robertsson 2017). Based on this we chose to include results only from Cox analyses. Bilateral THAs are dependent observations, but the influence of bilaterality on the outcome has been found to be negligible, also in the case of infection (Lie et al. 2004, Ranstam et al. 2011b, Dale et al. 2012). Hence, patients with bilateral THAs were included, and considered independent.

We calculated 95% CIs for survival rates and HRRs, and considered non-overlapping 95% CIs statistically significant. We used IBM SPSS 26.0 (IBM Corp, Armonk, NY, USA) and R statistical software packages for analyses (R Foundation for Statistical Computing, Vienna, Austria), and the study was performed in accordance with the STROBE and RECORD statements.

### Ethics, data sharing plan, funding, and potential conflicts of interests

The registration of data and the study was performed confidentially on patient consent and according to Norwegian and EU data protection rules. Data may be accessible upon application to the NAR. The study was fully financed by the NAR, and no conflict of interest is declared.

## Results

108,854 primary THAs in 91,621 patients met the inclusion criteria. 1,365 (1.3%) first revisions due to deep infection after primary THA were reported. Median follow-up was 5.4 (inter quartile range [IQR] 6.7) years, median age was 70 (IQR 14) years whereas mean ASA class for the THA patients was 2.0.

### Time trend of revision due to deep infection

**Figure 1.**
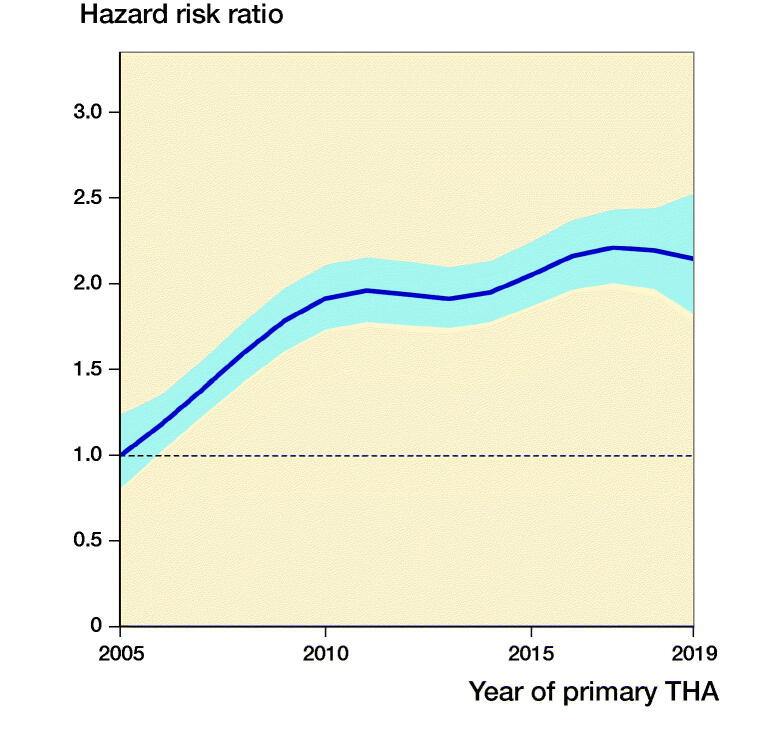
Relationship between year of primary surgery and risk of revision due to deep infection (with 95% confidence interval) for all THAs, adjusted for sex, age, ASA class, indication for primary THA, duration of surgery, surgical approach, and modularity of the THA. The broken line represents the HRR in 2005 (HRR = 1).

**Figure 2.**
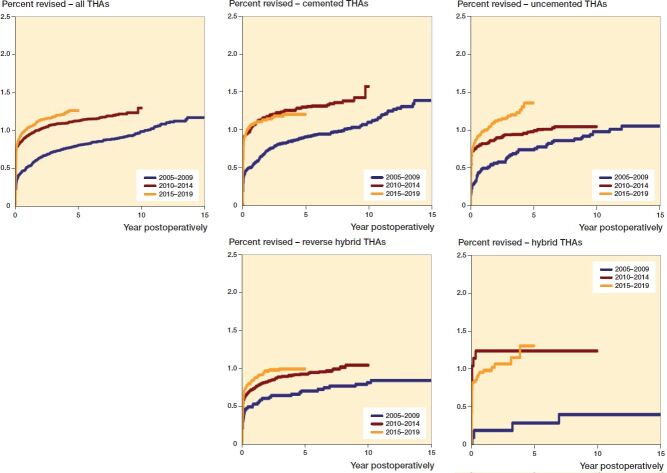
Curves of adjusted revision-percentage due to deep infection, for all THAs, for cemented THAs, uncemented THAs, reverse hybrid THAs, and hybrid THAs, for 3 periods of primary surgery, adjusted for sex, age, ASA class, indication for primary THA, duration of surgery, surgical approach, and modularity of the THA.

The annual number of revisions due to infection is presented in Table 1, whereas the annual increase in risk of revision due to infection presented in Figure 1. We found that the risk of revision due infection was higher for the periods 2010–2015 and 2015–2019 compared with 2005–2009. This finding was valid for all fixation methods. We found no difference between the 2 most recent periods, except for the cohort of uncemented THAs, in which there was an increased risk for revision due to infection in 2015–2019 (CI 1.4 [1.1–1.7]), compared with 2010–2014, as well (Figure 2 and Table 2, see Supplementary data).

**Table 1.** Absolute annual number of primary THAs and revisions due to infection for the period 2005–2019

Year of primary	Number of THAs revised due to infection					Number of primary
	0–30	31–90	91 days	1–5		
THA	days	days	–1 year	years	Total (%)	THAs
2005	6	4	8	15	46 (0.9)	5,849
2006	10	6	6	23	55 (1.2)	5,816
2007	23	9	4	22	74 (1.2)	6,099
2008	30	10	10	17	78 (1.4)	6,327
2009	34	12	13	17	95 (1.5)	6,577
2010	53	11	10	18	98 (1.6)	6,722
2011	71	8	7	17	111 (1.3)	6,783
2012	54	10	13	16	98 (1.3)	7,272
2013	58	11	11	14	96 (1.3)	7,228
2014	47	14	11	11	84 (1.1)	7,333
2015	60	17	14	n.a.	n.a.	7,705
2016	65	18	17	n.a.	n.a.	8,247
2017	64	15	20	n.a.	n.a.	8,539
2018	72	16	12	n.a.	n.a.	9,053
2019	n.a.	n.a.	n.a.	n.a.	n.a.	9,304

n.a. = not applicable due to incomplete follow-up

**Figure 3.**
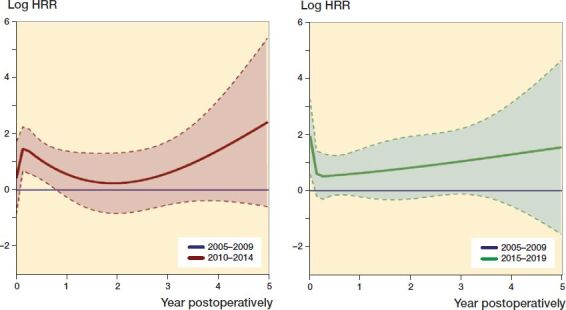
The relationship between HRR of revision due to infection and time­span postoperatively after primary THAs for the period 2010–2014 (red line) and 2015–2019 (green line) compared with 2005–2009 (blue lines). Smoothed Schoenfeld residuals adjusted for sex, age, ASA class, indication for primary THA, duration of surgery, surgical approach, and modularity of the THA (solid lines) with 95% confidence intervals (broken lines).

The increase in risk of revision due to infection was most pronounced in the first 30 postoperative days, but for 2015–2019 we found an increased risk for the whole first postoperative year (Figure 3 and Table 3, see Supplementary data).

Excluding THAs with metal-on-metal articulation (n = 300), THAs due to other causes than osteoarthritis, and monobloc THAs did not alter our findings. Restricting follow-up for each period to 0–5 years also showed similar results.

### Factors associated with risk of revision due to infection

The distribution of risk factors is presented in Table 4 (see Supplementary data). Patient-related factors such as age, sex, and indication for primary THA was stable throughout the period studied. There was more comorbidity in patients undergoing primary THA, from mean ASA class 1.8 (SD 0.7) in 2005 to 2.1 (SD 0.6) in 2019. The duration of surgery decreased slightly, the use of mono­bloc stems was terminated, and there was a shift towards uncemented fixation. Further, the use of posterolateral, anterolateral, and anterior surgical approaches increased at the expense of the direct lateral approach, which used to be the most common in our country.

We assessed the impact of the different risk factors adjusted for in the Cox analyses, and the findings are presented in Table 5 (see Supplementary data). Male sex, advanced age (> 75 years), and comorbidity (ASA class > 1) were patient-related factors associated with increased risk of revision due to infection. THA due to complications after hip fracture surgery and due to avascular necrosis of the femoral head was associated with an increased risk of revision due to infection, whereas THA due to complications after childhood hip disease was associated with a lower risk. Long duration of surgery (> 100 minutes), and anterolateral and lateral surgical approaches were associated with a slightly higher risk of revision due to infection.

Uncemented and hybrid THAs had higher risk of revision due to infection than cemented THAs, whereas reverse hybrid THAs had lower risk. Compared with modular THAs, monobloc THAs apparently had half the risk of revision due to infection, as expected, since debridement of these were not reported to the NAR as a revision, as explained earlier.

Trends that may have contributed to the increased risk of revision for infection were a higher number of patients with substantial comorbidity, and more use of uncemented and hybrid THA (Tables 4 and 5, see Supplementary data). Trends that may have contributed to less increase in risk of revision for infection were less use of the lateral surgical approach and shorter duration of surgery (Tables 4 and 5, see Supplementary data).

### Time trend of revision due to aseptic loosening

The risk of revision due to aseptic loosening, compared with 2005–2009, was decreasing, with 0.6 (CI 0.5–0.7) for 2010–2014, and 0.8 (CI 0.7–1.0) for 2015–2019 respectively.

## Discussion

Our main finding was an increased risk for revision due to deep infection after primary THA for the 2 consecutive 5-year periods after 2005–2009. The most pronounced increase was for THAs performed before 2010, whereafter the risk of revision due to infection seems to have levelled out.

Our findings confirm the trend from earlier studies from the NAR and the Nordic Arthroplasty Register Association (NARA) on the risk of revision due to infection (Dale et al. 2009, 2012). There are also other studies reporting an increased risk of PJI (Lenguerrand et al. 2017, Brochin et al. 2018, Kurtz et al. 2018). The finding that the increase in PJI is flattening out after 2010 is in concordance with findings from New York State (Perfetti et al. 2017).

On the other hand, several infection surveillance registries report a trend of decreasing rates of surgical site infection (SSI) after THA, including both superficial and deep infections (Manniën et al. 2008, Choi et al. 2016, Sodhi et al. 2019). This was also the finding of the Norwegian Institute of Public Health’s SSI surveillance registry (Berg et al. 2019). The European Centre for Disease Prevention and Control (ECDC) reports a stable in-hospital incidence of SSI after total hip arthroplasty since 2011 (ECDC 2019), similar to what we found for that period. A review from 2015 reports increasing risk of SSI in several countries (Lamagni et al. 2015). This variety in trends might be explained by the differences in definitions and duration of observation between SSIs reported in regional or national surveillance systems, and revision due to infection, as reported to the arthroplasty registers. SSI is observed at discharge from hospital or at post-discharge surveillance by a general physician (30 days, 90 days, or 1 year postoperatively), in concordance with a specific set of diagnostic criteria and strict definition, and may be superficial or deep (ECDC 2017). In Norway, we have only 30 days’ surveillance of SSI after THA, but with good completeness since 2013 (Berg et al. 2019). In the NAR, however, the surgeon reports revision due to infection at any time after the primary THA. In our material, we found stable risk of revision due to infection in the first 30 days postoperatively for the period 2010–2019 (Table 3), in contrast to the slightly reduced incidence of SSIs from the Norwegian SSI surveillance program in 2013–2018 (Berg et al. 2019). Even if we do not have absolute numbers for direct comparison, and the definitions of infection are different, this may reflect a trend towards more revisions being performed due to superficial SSIs and prolonged wound drainage, which would, in that case, result in an apparent increase in risk of infection in our material.

Surgeons may report to the NAR debridement for prolonged wound drainage as revision due to infection. This revision strategy has evolved because prolonged wound drainage and superficial SSIs are strongly associated with PJI (Zhu et al. 2015). Some of these cases will have negative cultures and will not fulfill criteria for a PJI. However, they remain registered as infections in the NAR. This may explain the discrepancies between the 2 registers, at least to some degree.

For 2015–2019, we found an increased risk of revision due to infection in the first postoperative year. In contrast to our earlier findings, there was also an increased risk of revision later than 90 days postoperatively, which indicates a prolonged increase in risk of revision due to infection in recent years (Dale et al. 2012).

The finding that the risk of revision increased more for the uncemented THAs compared with the other fixation methods may be explained by more frequent use of uncemented THAs in older patients with more comorbidity in recent years. The increased use of uncemented THA was most pronounced in 2005–2009, and as uncemented THA was found to be a risk factor associated with revision due to infection, this may partly explain our finding of increased risk of revision due to infection during this period. All patients with cemented components in the present study had antibiotic-loaded bone cement. This has been found to be beneficial as prophylaxis against postoperative infection (Parvizi et al. 2008, Dale et al. 2012, Wang et al. 2013). Hybrid THAs, although few, had higher risk of revision due to infection compared with cemented THAs. This is in concordance with our previous and others’ findings (Pedersen et al. 2010b, Dale et al. 2012, Leong et al. 2020).

Compared with our previous study, we now had the benefit of being able to adjust for ASA class. However, this did not change the finding of increased risk of revision due to infection. Even if risk factors for revision due to infection may differ from risk factors for PJI, one possible explanation for the increased risk of infection is that THA is now being performed in frailer patients. We found an increase in mean ASA class for THA patients, and that ASA 2 and higher was associated with revision due to infection. The change in ASA class for THA patients was most pronounced between 2005–2009 and 2010–2014. Some comorbidities, such as obesity and diabetes with hyperglycemia, are found to be potent risk factors for postoperative infection and have an increasing incidence in the population (Pedersen et al. 2010a, Jämsen et al. 2012, Lamagni et al. 2015, Liu et al. 2015). These specific risk factors are not reported to the NAR, and might, if in our material as in the general population the prevalence increases, and if not fully captured by ASA class, contribute to an increased risk of infection.

In the present study primary THA for an acute hip fracture was more used in 2015–2019 than in 2005–2009. Acute hip fracture was not found to be a risk factor for revision due to infection. Displaced femoral neck fractures are recommended to be treated with hemiarthroplasty in old and frail patients with limited life expectancy (Gjertsen 2019). However, in patients expected to live longer, THA may the recommended treatment in certain cases. This selection of THA for healthier patients may explain why acute hip fracture was not found to be a risk factor for revision due to infection, as may have been expected.

There have been improvements in diagnostic procedures of PJI, and more standardized sampling, culturing, and analyzing techniques lead to fewer samples being false negative (Parvizi et al. 2016, Ting and Della Valle 2017). In addition, bacteria like *Staphylococcus epidermidis* and *Cutibacterium acnes* have emerged as important agents of implant infection (Zeller et al. 2018, Flurin et al. 2019). This may have resulted in more low-grade infections being correctly diagnosed, revised, and reported, where such infections earlier may have been overlooked or misdiagnosed as aseptic loosening.

We found a trend towards more use of surgical approaches associated with lower risk of revision due to infection, and shorter duration of surgery. This should be considered beneficial and contribute to lower risk of revision due to infection as found by previous publications (Dale et al. 2009, Amlie et al. 2014, Mjaaland et al. 2017, Miller et al. 2018).

Registers can provide a useful source of information on incidences and trends because of large numbers and long duration of observation. The NAR contain good quality, detailed information on THA patients and primary and revision surgery, gathered uniformly over a long period. Our data are prospective and with acceptable completeness on possible risk factors for primary and revision THA (Furnes et al. 2019). We therefore have an excellent base for a trend study on a relatively rare complication like PJI. Since a large number of THAs from a nationwide population were included, external validity is expected to be good.

Register studies do have, however, inherent limitations (Varnum et al. 2019). Even if we adjusted for several important factors that could be associated with revision due to infection, there will be residual confounding. Such factors may be changes over time in reporting, revision threshold, diagnostics, surgeon awareness, selection of patients, and the virulence and resistance of bacteria causing infection. These factors may only partly be elucidated in a national arthroplasty register.

Reported THA revision rates due to infection are not necessarily the same as rates of PJI, although we have reason to believe that this is a close approximation, since guidelines recommend revision in the case of suspected infection, and reporting of revisions to the NAR is good. Improved reporting of revision due to infection may explain our findings to some degree. However, compared with validation studies from Sweden and Denmark, and considering the 93% reporting of revisions to the NAR, our reporting of revision due to infection resembles the “true” incidence of PJI reported from similar registers (Lindgren et al. 2014, Gundtoft et al. 2015, Furnes et al. 2019). Yet another limitation is that an erroneous diagnosis of infection is not corrected in the NAR, when results from peroperative bacterial sampling presents. This may lead to both under-reporting of low-grade infections and over-reporting in the case of negative bacterial cultures. Misreporting will only influence our findings if it changes during the period studied. Based on the findings of a slight decrease in risk of revision due to aseptic loosening and the fact that there may have been improvements in diagnostics and surgeons’ awareness, there may have been a decrease in misreporting.

Focus on the importance of thorough reporting has probably improved the reporting of revisions due to infection over the period studied. However, a time trend evaluation of this has not been performed.

In summary, our finding of increased risk of revision is probably multifactorial. Partly it may reflect changes over time in reporting, revision threshold, diagnostics, surgeon awareness, surgical volume and skills, and the virulence and resistance of bacteria causing infection. An increase in the risk of revision due to infection in such cases will not reflect an increase in the incidence of PJI.

On the other hand, if THA is performed on patients with more comorbidity, higher age, or implants or techniques associated with a higher risk of infection are used, this would, as found in our study, contribute to an increased risk of infection after THA.

To conclude, the risk of revision due to infection after THA increased by approximately 50% through the period 2005–2019. However, the increase in risk of revision due to infection appears to have levelled out after 2010. The increase was mainly caused by more revisions during the first postoperative year. Uncemented THAs were increasingly used during the study period, and also in patients with comorbidity, and we found a corresponding increase in risk of revision due to infection for uncemented THAs. In addition, THA patients had more comorbidity within all groups of fixation, and this may have contributed to the increased risk of revision due to infection.

## Supplementary Material

Supplemental Material

## Supplementary data

Tables 2–5 are available as supplementary data in the online version of this article, http://dx.doi.org/10.1080/17453674.2020.1851533
