# Supplementary material for: Increasing but levelling out risk of revision due to infection after total hip arthroplasty: a study on 108,854 primary THAs in the Norwegian Arthroplasty Register from 2005 to 2019
Source: Acta Orthop. 2020 Nov 24;92(2):208–14. doi: 10.1080/17453674.2020.1851533 (PMC8158216; doi:10.1080/17453674.2020.1851533)
Supplement: Supplemental Material [file IORT_A_1851533_SM9323.pdf]

# Supplementary data

**Table 2. Hazard rate ratios (HRR) and 5-year revision rate estimates, for revision due to deep infection, with number of primary THAs included and number of revisions due to deep infection reported, for the 3 time periods and the 4 fixation methods**

| THA fixation Period | No. of THAs | Number revised [a] | HRR (CI) | p-value | 5-year follow-up Number revised [a] | Adjusted revision rate (CI) | At risk after 5 years |
|---|---|---|---|---|---|---|---|
| All | | | | | | | |
| 2005–2009 | 30,668 | 348 | 1 | | 279 | 0.8 (0.7–0.9) | 29,740 |
| 2010–2014 | 35,338 | 487 | 1.4 (1.2–1.6) | < 0.001 | 465 | 1.2 (1.1–1.3) | 34,130 |
| 2015–2019 | 42,848 | 530 | 1.6 (1.3–1.9) | < 0.001 | 530 | n.a. | 32,334 |
| Cemented | | | | | | | |
| 2005–2009 | 20,031 | 237 | 1 | | 190 | 0.9 (0.8–1.1) | 19,389 |
| 2010–2014 | 11,478 | 188 | 1.4 (1.2–1.7) | 0.001 | 180 | 1.4 (1.2–1.6) | 11,022 |
| 2015–2019 | 11,263 | 144 | 1.5 (1.1–1.9) | 0.002 | 144 | n.a. | 8,534 |
| Uncemented | | | | | | | |
| 2005–2009 | 5,008 | 63 | 1 | | 48 | 0.8 (0.5–1.0) | 4,858 |
| 2010–2014 | 9,495 | 121 | 1.2 (0.7–1.7) | 0.3 | 116 | 1.1 (0.9–1.3) | 9,192 |
| 2015–2019 | 16,530 | 392 | 1.6 (1.2–2.3) | 0.005 | 372 | n.a. | 11,964 |
| Reverse hybrid | | | | | | | |
| 2005–2009 | 5,026 | 44 | 1 | | 38 | 0.7 (0.5–1.0) | 4,909 |
| 2010–2014 | 13,660 | 165 | 1.3 (0.9–1.9) | 0.1 | 156 | 0.9 (0.8–1.1) | 13,244 |
| 2015–2019 | 11,464 | 125 | 1.5 (1.0–2.2) | 0.04 | 125 | n.a. | 9,481 |
| Hybrid | | | | | | | |
| 2005–2009 | 603 | 4 | 1 | | 3 | 0.3 (0.0–3.1) | 584 |
| 2010–2014 | 705 | 13 | 3.6 (1.1–12) | 0.04 | 13 | 1.4 (0.1–13) | 672 |
| 2015–2019 | 3,591 | 53 | 3.6 (1.1–12) | 0.04 | 53 | n.a. | 2,355 |

[a] Due to infection.
The HRR and revision estimates are adjusted for sex, age, ASA class, indication for primary THA, duration of surgery, surgical approach, and modularity of the THA. HRRs are given with 95% confidence intervals (CI).
n.a. = not applicable due to incomplete 5-year follow up.

**Table 3. HRR with 95% confidence intervals for revision due to infection relative to timespan postoperatively, for the 3 time periods**

| Period | THAs overall | Revised [a] overall | 0–30 days Revised [a] | 0–30 days HRR (CI) | 31–90 days Revised [a] | 31–90 days HRR (CI) | 91 days–1 year Revised [a] | 91 days–1 year HRR (CI) | 1–5 years Revised [a] | 1–5 years HRR (CI) | THAs at risk after 5 years |
|---|---|---|---|---|---|---|---|---|---|---|---|
| 2005–2009 | 30,668 | 348 | 103 | 1 | 41 | 1 | 41 | 1 | 94 | 1 | 20,173 |
| 2010–2014 | 35,338 | 487 | 283 | 2.2 (1.8–2.8) | 54 | 1.0 (0.7–1.6) | 52 | 1.1 (0.7–1.8) | 76 | 0.7 (0.5–1.0) | 26,076 |
| 2015–2019 [b] | 42,848 | 530 | 327 | 2.3 (1.8–2.9) | 83 | 1.6 (1.0–2.5) | 72 | 1.6 (1.0–2.6) | 48 | 0.9 (0.6–1.3) | 25,214 |

[a] Due to infection.
[b] The number revised due to infection will be an underestimate due to incomplete 5-year follow-up.
The HRR and revision estimates are adjusted for sex, age, ASA class, indication for primary THA, duration of surgery, surgical approach, and modularity of the THA. HRRs are given with 95% confidence intervals.

**Table 4. Distribution of patient and surgery related factors, in the 3 periods**

| Risk factor | No. of THAs | Distribution of factors (%) 2005–2009 | 2010–2014 | 2015–2019 |
|---|---|---|---|---|
| Total number | 108,854 | 30,668 | 35,338 | 42,848 |
| Sex | | | | |
| Male | 37,710 | 33 | 35 | 36 |
| Female | 71,144 | 67 | 65 | 64 |
| Age | | | | |
| < 45 | 3,490 | 3 | 3 | 3 |
| 45–54 | 8,909 | 7 | 8 | 9 |
| 55–64 | 24,981 | 23 | 23 | 22 |
| 65–74 | 39,144 | 33 | 36 | 38 |
| 75–84 | 27,258 | 28 | 25 | 23 |
| ≥ 85 | 5,072 | 5 | 5 | 5 |
| ASA class | | | | |
| 1 | 20,682 | 29 | 16 | 14 |
| 2 | 66,407 | 52 | 64 | 65 |
| 3 | 21,318 | 19 | 19 | 20 |
| 4 | 447 | 0.4 | 0.3 | 0.5 |
| Indication for primary THA | | | | |
| Osteoarthritis | 83,770 | 76 | 77 | 77 |
| Inflammatory hip disease | 2,346 | 3 | 2 | 2 |
| Acute hip fracture | 3,769 | 2 | 3 | 5 |
| Complications after | | | | |
| hip fracture | 5,743 | 7 | 5 | 4 |
| childhood hip disease | 9,624 | 9 | 9 | 8 |
| Avascular necrosis of the femoral head | 2,888 | 3 | 3 | 3 |
| Other | 714 | 1 | 1 | 1 |
| Duration of surgery | | | | |
| < 70 minutes | 29,709 | 18 | 27 | 34 |
| 70–99 minutes | 46,287 | 44 | 42 | 41 |
| 100–129 minutes | 24,126 | 27 | 22 | 18 |
| ≥ 130 minutes | 8,732 | 10 | 8 | 6 |
| Surgical approach | | | | |
| Anterior | 5,979 | 1 | 6 | 8 |
| Anterolateral | 8,247 | 6 | 12 | 14 |
| Lateral | 47,048 | 67 | 50 | 12 |
| Posterolateral | 47,580 | 25 | 32 | 66 |
| Modularity of THA | | | | |
| Monobloc | 3,936 | 11 | 2 | 0 |
| Modular | 104,918 | 89 | 98 | 100 |
| Fixation principle | | | | |
| Cemented | 42,772 | 65 | 32 | 26 |
| Uncemented | 31,033 | 16 | 27 | 39 |
| Reverse hybrid | 30,150 | 16 | 39 | 27 |
| Hybrid | 4,899 | 2 | 2 | 8 |

**Table 5. Number of primary THAs included and number of reported first revisions due to deep infection, and the association between potential risk factors and revision due to infection**

| Risk factor | No. of THAs | Number revised [a] | HRR (CI) | Adjusted 5-year revision rate [a] (CI) |
|---|---|---|---|---|
| Total number | 108,854 | 1,365 | | |
| Sex | | | | |
| Male | 37,710 | 710 | 2.1 (1.9–2.3) | 1.7 (1.6–1.9) |
| Female | 71,144 | 655 | 1 | 0.8 (0.8–0.9) |
| Age | | | | |
| < 45 | 3,490 | 45 | 1.1 (0.8–1.6) | 1.1 (0.7–1.5) |
| 45–54 | 8,909 | 86 | 0.9 (0.7–1.1) | 0.9 (0.7–1.1) |
| 55–64 | 24,981 | 289 | 1.0 (0.9–1.2) | 1.0 (0.9–1.2) |
| 65–74 | 39,144 | 452 | 1 | 1.0 (0.9–1.1) |
| 75–84 | 27,258 | 397 | 1.2 (1.1–1.4) | 1.2 (1.1–1.4) |
| ≥ 85 | 5,072 | 96 | 1.5 (1.2–1.9) | 1.5 (1.2–1.8) |
| ASA class | | | | |
| 1 | 20,682 | 160 | 1 | 0.7 (0.6–0.8) |
| 2 | 66,407 | 770 | 1.5 (1.3–1.8) | 1.1 (1.0–1.2) |
| 3 | 21,318 | 422 | 2.3 (1.9–2.8) | 1.6 (1.4–1.8) |
| 4 | 447 | 13 | 3.3 (1.8–5.8) | 2.4 (1.0–3.7) |
| Indication for primary THA | | | | |
| Osteoarthritis | 83,770 | 1,019 | 1 | 1.1 (1.0–1.2) |
| Inflammatory hip disease | 2,346 | 38 | 1.3 (0.9–1.8) | 1.3 (0.9–1.7) |
| Acute hip fracture | 3,769 | 53 | 1.1 (0.8–1.4) | 1.1 (0.8–1.4) |
| Complications after | | | | |
| hip fracture | 5,743 | 104 | 1.2 (1.0–1.5) | 1.3 (1.0–1.5) |
| childhood hip disease | 9,624 | 78 | 0.7 (0.6–0.9) | 0.8 (0.6–1.0) |
| Avascular necrosis of the femoral head | 2,888 | 60 | 1.5 (1.1–1.9) | 1.8 (1.3–2.3) |
| Other | 714 | 13 | 1.1 (0.6–2.0) | 1.7 (0.6–2.7) |
| Duration of surgery | | | | |
| < 70 minutes | 29,709 | 330 | 1.0 (0.8–1.1) | 1.0 (0.9–1.1) |
| 70–99 minutes | 46,287 | 547 | 1 | 1.0 (0.9–1.1) |
| 100–129 minutes | 24,126 | 329 | 1.1 (1.0–1.3) | 1.2 (1.0–1.3) |
| ≥130 minutes | 8,732 | 159 | 1.4 (1.2–1.7) | 1.4 (1.2–1.7) |
| Surgical approach | | | | |
| Anterior | 5,979 | 52 | 0.8 (0.6–1.1) | 0.7 0.5–1.0) |
| Anterolateral | 8,247 | 81 | 1.0 (0.8–1.2) | 0.9 (0.7–1.1) |
| Lateral | 47,048 | 683 | 1.4 (1.2–1.6) | 1.4 (1.3–1.5) |
| Posterolateral | 47,580 | 549 | 1 | 0.9 (0.8–1.0) |
| Modularity of THA | | | | |
| Monobloc | 3,936 | 28 | 0.5 (0.3–0.7) | 0.6 (0.4–0.8) |
| Modular | 104,918 | 1,337 | 1 | 1.1 (1.0–1.2) |
| Fixation principle | | | | |
| Cemented | 42,772 | 569 | 1 | 1.0 (0.9–1.2) |
| Uncemented | 31,033 | 392 | 1.2 (1.0–1.4) | 1.3 (1.1–1.4) |
| Reverse hybrid | 30,150 | 334 | 0.8 (0.7–1.0) | 0.9 (0.8–1.0) |
| Hybrid | 4,899 | 70 | 1.3 (1.0–1.6) | 1.3 (1.0–1.7) |

Adjusted revision rates and HRR estimates for sex, age, ASA class, indication for primary THA, duration of surgery, surgical approach, modularity of the THA, and fixation method. The HRRs are presented with 95% confidence intervals. The risk factors are adjusted for all the other risk factors in addition to year of primary surgery to assess the association with revision due to infection.
